# Development of Terahertz Imaging Markers for Pancreatic Ductal Adenocarcinoma using Maximum *A Posteriori* Probability (MAP) Estimation

**DOI:** 10.1021/acsomega.2c07080

**Published:** 2023-03-08

**Authors:** Debamitra Chakraborty, Bradley N. Mills, Jing Cheng, Ivan Komissarov, Scott A. Gerber, Roman Sobolewski

**Affiliations:** † Materials Science Graduate Program, University of Rochester, Rochester, New York 14627-1299, USA; ‡ Laboratory for Laser Energetics, University of Rochester, Rochester, New York 14623, USA; § Department of Surgery, University of Rochester Medical Center, Rochester, New York 14642, USA; ∥ Department of Electrical and Computer Engineering and Department of Physics, University of Rochester, Rochester, New York 14627-0231, USA

## Abstract

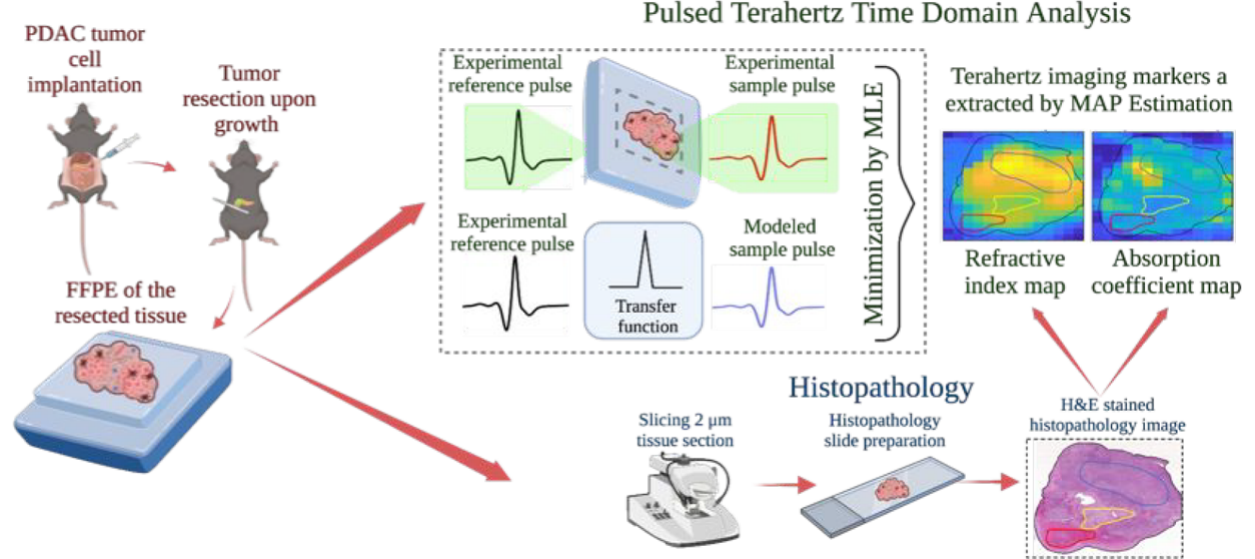

Pancreatic ductal adenocarcinoma (PDAC) is one of the significant reasons for cancer-related death in the United States due to a lack of timely prognosis and the poor efficacy of the standard treatment protocol. Immunotherapy-based neoadjuvant therapy, such as stereotactic body radiotherapy (SBRT), has shown promising results compared to conventional radiotherapy in strengthening the antitumor response in PDAC. To probe and quantify the antitumor response with SBRT, we propose to study the tumor microenvironment using terahertz time-domain spectroscopy (THz-TDS). Since the tumor’s complex microenvironment plays a key role in disease progression and treatment supervision, THz-TDS can be a revolutionary tool to help in treatment planning by probing the changes in the tissue microenvironment. This paper presents THz-TDS of paraffin-embedded PDAC samples utilizing a clinically relevant genetically engineered mouse model. This Article aims to develop and validate a novel time-domain approximation method based on maximum *a posteriori* probability (MAP) estimation to extract terahertz parameters, namely, the refractive index and the absorption coefficient, from THz-TDS. Unlike the standard frequency-domain (FD) analysis, the parameters extracted from MAP construct better-conserved tissue parameters estimates, since the FD optimization often incorporates errors due to numerical instabilities during phase unwrapping, especially when propagating in lossy media. The THz-range index of refraction extracted from MAP and absorption coefficient parameters report a statistically significant distinction between PDAC tissue regions and their healthy equivalents. The coefficient of variation of the refractive index extracted by MAP is one order of magnitude lower compared to the one extracted from FD analysis. The index of refraction and absorption coefficient extracted from the MAP are suggested as the best imaging markers to reconstruct THz images of biological tissues to reflect their physical properties accurately and reproducibly. The obtained THz scans were validated using standard histopathology.

## 1 Introduction

Pancreatic ductal adenocarcinoma (PDAC) is an aggressively progressive malignancy that contributes to the third-highest number of cancer deaths in the United States.^1^ The total five-year survival rate is 9% due to the absence of timely diagnosis and inadequate response to conventional treatment procedures such as surgery, chemotherapy, and radiation therapy.^2^ More than 80% of PDAC patients are diagnosed with the advanced stage of the disease often after metastasis and, at the time of diagnosis, there are no curative treatments.^2,3^ The currently available serum biomarkers, like CA19-9, are insufficient to detect pancreatic cancer at an early stage due to their low specificity and sensitivity. At diagnosis, merely 20% of the patients qualify for a potentially curative pancreatectomy, and most patients, nevertheless, experience lethal recurrence and/or systemic metastases regardless of second-line intervention.^4^ The existing standard chemotherapy with “gemcitabine” has very narrow efficiency, extending the patient’s overall survival by only 6–12 weeks.^4^ This poor performance of current standard treatments is often associated with the lack of understanding of the disease’s complex biology, especially its heterogeneous microenvironment. Alternatively, immunotherapy approaches that often employ immune-priming neoadjuvants, such as stereotactic body radiotherapy (SBRT), aim to stimulate immunogenic tumor cell death and thereby educate and amplify the antitumor immune response.^5^ However, tumor cells are capable of mitigating DNA damage to escape cell death mechanisms, leading to both interpatient and intratumoral response variabilities. We postulate that the terahertz (THz) imaging technique can serve as a novel approach for mapping and measuring the cytotoxic responsivity of PDAC to neoadjuvant therapies, such as SBRT. Hence, probing the treated PDAC microenvironment using THz imaging can help us to investigate whether the tumor is responding or likely to respond to a given therapy. This information can then be used to assess the efficacy of neoadjuvant or first-line therapy and subsequently inform adjuvant and/or second-line treatment approaches. Currently, comprehensive tumor characterization requires multiplexed immunohistochemistry assays that are expensive and time-consuming given the large number of molecular targets needed to identify and validate each tumor property.^6^ Instead, THz time-domain spectroscopy (THz-TDS) based imaging can be performed to acquire the same level of information at a fraction of the cost. Additionally, pseudoprogression is an atypical therapeutic response pattern that is commonly observed following immunotherapy. This phenomenon is characterized by an initial observation of post-treatment tumor progression followed by a decrease in tumor burden, which often leads to the premature discontinuation of therapy. The lack of standardized criteria for evaluating immunotherapy responses has made pseudoprogression a significant challenge for physicians.^7^ Terahertz imaging holds the potential to serve as an *ex vivo* imaging platform that objectively maps tumor responses to immunotherapy. Further development of this technology could provide a tool that deconvolutes tumor-specific treatment responses independent of pseudoprogression-related features much earlier in the therapeutic window relative to traditional pathologic examination.

According to the literature reviewed above, there is a crucial demand to develop new technologies with a novel imaging marker competent for probing and identifying the complex tumor microenvironment. Hence, THz time-domain spectroscopy (THz-TDS) can be applied to probe the heterogeneity in the PDAC microenvironment. The THz-TDS technique is a label-free, noninvasive, and nonionizing tool for biomedical imaging, especially in tumor prognosis. THz radiation lacks the energy to break chemical bonds or ionize molecules/atoms in studied tissues, making it harmless to living organisms, in contrast to much higher energy photons such as ultraviolet light or, especially, X rays.^8,9^ THz-TDS provides submillimeter spatial resolution and molecular fingerprinting by providing both the frequency and time-domain information and could be capable of detecting pancreatic cancers.

**Figure 1:**
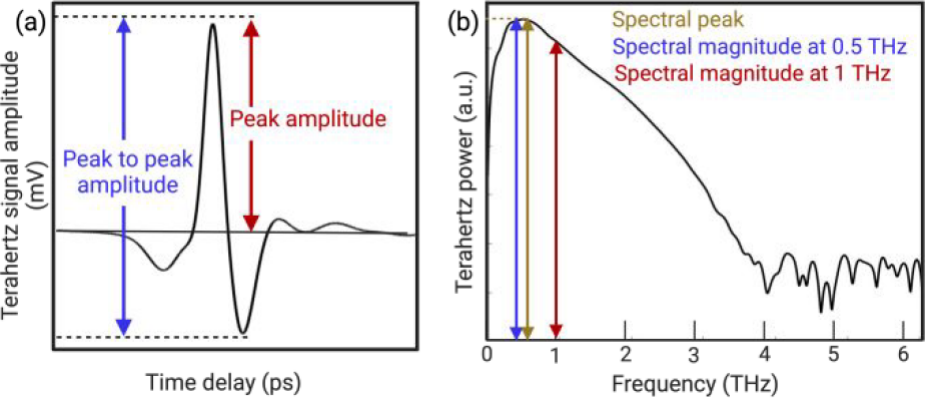
Representative (a) pulsed TD signal and (b) corresponding FD spectrum obtained from the TD pulse via the FFT method. The conventional features used as imaging markers corresponding to the signal and spectrum are indicated. Created with BioRender.com, agreement no. LG24VWZ220.

Extensive bioimaging research based on the THz-TDS technique has been conducted on various types of malignancy, both *in vitro* and *ex vivo*, with reports revealing significant differences in the tissue properties in tumors as compared to their healthy controls (referred in the literature to as a normal tissue).^10^ However, despite the availability of vast and rich literature, all these studies lack standardized THz imaging markers to map out the tissue properties. Presently, there are mainly two approaches to report the difference between the tumor and normal tissues; the first is to map an image of time- or frequency-domain signal amplitudes measured at corresponding locations, *i.e.*, the maximum amplitude of the signal from the tumor sample, normalized with respect to a reference (either an empty setup or a healthy control) and the second is reporting a difference in the tissue’s optical parameters like the refractive index *n* and/or the absorption coefficient *α* in the THz frequency region.^8,11−18^Figure 1 presents a collection of features mapped in literature as imaging markers obtained from an experimentally acquired THz TD pulse and its corresponding frequency spectrum obtained by fast Fourier transform (FFT). The lack of standardized, optimal imaging markers prevents reliable comparisons of data reported by different research groups. Since subtle changes in the tissue microenvironment can markedly impact material properties, there is a need for THz imaging markers that are unbiased and reproducible.

This work aims to propose an optimized set of imaging markers extracted by a technique based on maximum *a posteriori* probability (MAP) estimation from pulsed THz time-domain data for murine tissue with PDAC. The differences in optical parameters in the THz region between different areas within the tissue could be attributed to heterogeneity in the tissue constituents. THz spectroscopy has demonstrated detectable contrasts in many freshly harvested biological tissues that can be attributed to differences in water content. Conversely, the formalin-fixed paraffin-embedded (FFPE) tissues that this study interrogates in the absence of water also show detectable THz contrast.^13^ We hypothesize that the presence of collagen and its variational density are major contributors to the observed contrasts in the microenvironments of FFPE PDAC tissues. However, an exact correlation of collagen density to THz extracted parameters necessitate experiments on engineered hydrogel phantom models of soft tissues^19^ and is beyond the scope of the presented work.

Since optical parameters can be the crucial markers for image reconstruction and understanding light-tissue interactions, accurately measuring them is necessary to provide reliable optical images at the THz range and their biophysical interpretation. In addition, the differences in imaging markers between normal and malignant tissues can be used to make a clinical diagnosis. Hence, efficient THz imaging markers capable of probing the tissue microenvironment can not only shed new light on the tumor prognosis but also can help in its treatment planning. In this work, we use PDAC as a case study, but the presented approach could be used for any tumor characterized using a THz-TDS technique. In this Article, we report the first experimental evidence of THz-TDS probing PDAC tissues using time-resolved MAP estimation to reconstruct THz optical parameters. We refer to the THz refractive index and absorption coefficient as imaging markers that can delineate tissue heterogeneity within the PDAC microenvironment.

The paper is organized as follows: section 2 describes our experimental THz-TDS system, followed by PDAC sample preparation, and MAP vs FD data analysis comparison. Section 3 presents our experimental results in the forms of transverse and longitudinal line scans and two-dimensional raster scans, as well as both qualitative and quantitative comparisons of the proposed imaging markers between MAP and FD methods. Finally, section 4 brings the discussion and section 5 conclusions.

## 2 Experimental Methods, Materials, and Data Analysis

### 2.1 THz-TDS Experimental Set-Up

**Figure 2:**
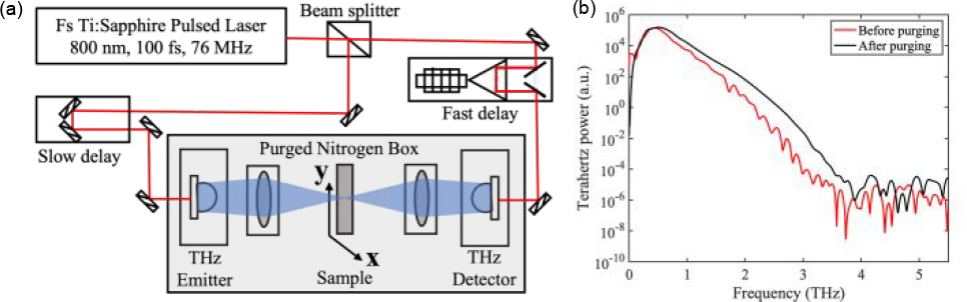
(a) Schematic diagram of our experimental setup. (b) THz FFT spectra of the empty setup (used as reference) before (red line) and after (black line) dry nitrogen purging, which is used to remove the spectral features of atmospheric water vapor.

Figure 2(a) presents a schematic diagram of the THz-TDS system used for all measurements in this work. A commercial mode-locked Ti:sapphire laser generates a train of 100 fs wide 800 nm wavelength optical pulses that is split by a 50:50 power ratio beam splitter into pump and probe beams. The pump beam, after passing a slow delay line, excites a commercial low-temperature-grown GaAs (LT-GaAs) THz emitter, while the probe beam is directed toward a commercial LT-GaAs detector antenna. Both the emitter and detector devices are equipped with hyper-hemispherical Si lenses to collimate the emitted THz beam and, subsequently, converge it into the detector. Two additional 1 cm focal length Teflon lenses are placed in front of the emitter and detector, respectively, to focus the THz signal into the sample under investigation and collect the transmitted one towards the detector. Note that in the probe beam path, we implemented a TRS-16 THz registering system from TeraVil.^20^ This fast optical delay line enables the direct collection of THz TD signals instead of using a conventional slow stepper motor-controlled delay line and a lock-in amplifier. The TRS-16 allows us to minimize the signal acquisition time to approximately 30 s per pixel while acquiring an average of 1000 traces with 1.778 fs time resolution for each collected time-resolved signal to obtain a signal-to-noise ratio of 86.24 dB. The overall image acquisition time increases with the decrease in the number of step-size and the number of signal averages. The TRS-16 controller simultaneously displays the TD waveform and the corresponding FFT spectrum. We perform all experiments inside a purged box made of Plexiglas with a constant dry-nitrogen flow to remove any spurious atmospheric water absorptions from the measured spectrum [see Figure 2(b), black line]. As indicated in Figure 2(a), our samples are studied in a transmission geometry by placing them at the THz radiation focal point between the emitter and detector on a stepper-motor-controlled *x*–*y* stage to perform scans. Before each tissue scan, we always run an empty setup test (without any sample) and use this result as a reference.

### 2.2 Sample Preparation

**Figure 3:**
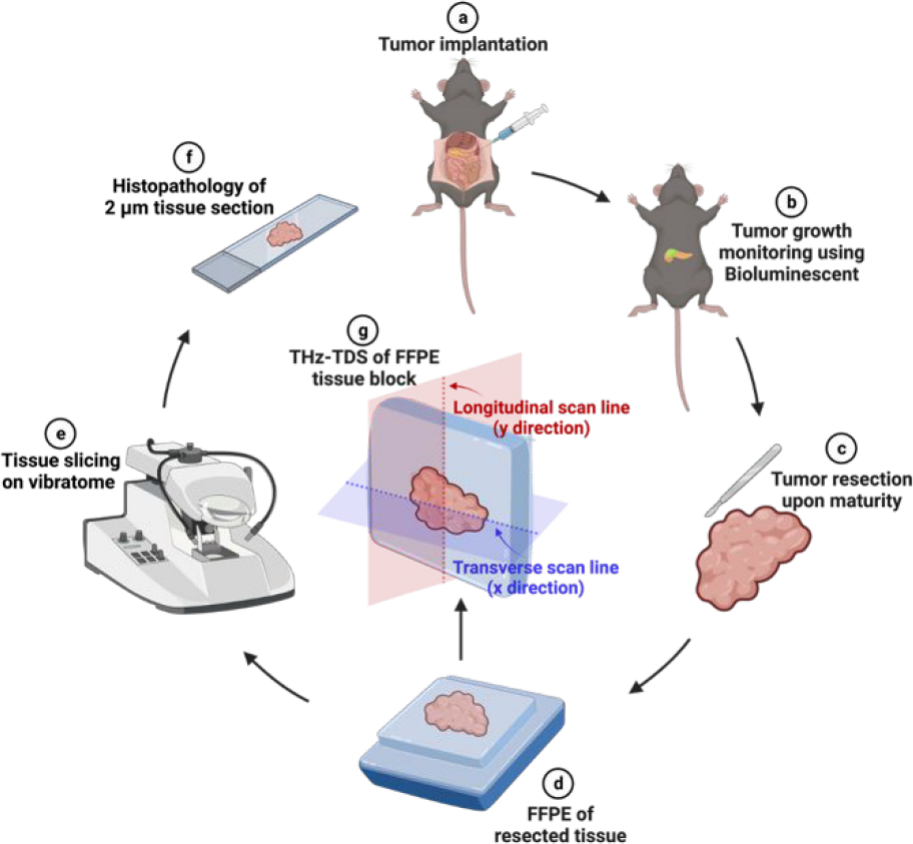
Outline of the experimental design. (a) Tumor cells are injected in the pancreatic tail. (b) The growth of the tumor is monitored using bioluminescent imaging. (c) The tumor is resected after it’s maturity. (d) The resected tumor is then formalin-fixed and paraffin-embedded for further measurement. (e) A 2 μm thin tissue is sliced on a vibratome. (f) This tissue section is used for histopathology. (g) About 4 mm thick FFPE tissue is removed from the cassette for THz-TDS. Line scans are performed along transverse (*x*-direction) and longitudinal (*y*-direction) axes, forming a cross-section. Created with BioRender.com, agreement no. TV24VWYQAH.

To create clinically relevant PDAC tumors for this investigation, we use the LSL-KrasG12D/+Trp53L/LPtf1a-Cre (KPC) GEMM, which generates tumors that closely reflect the diverse microenvironment of the human disease. The KPC model is the gold standard for preclinical PDAC research.^21^Figure 3 provides a detailed outline of the sample preparation and experimental design. We orthotopically implant KCKO pancreatic cancer cell lines stably expressing firefly luciferase (KCKO-luc) from the C57BL/6J background into mice.^2^ We anesthetize using an isoflurane anesthetic vaporizer (Scivena Scientific) and then we make a 10 mm laparotomy incision to expose the spleen and pancreas. Next, we suspend the cells in a 1:1 PBS/Matrigel (BD Biosciences) solution to inject 100 000 cells/mouse (100 mL) into the pancreatic tail. For 1 min after tumor cell injection, we put a cotton swab over the injection location to avoid peritoneal leakage and use IVIS bioluminescent imaging to monitor tumor growth and determine maturity. After the development of mature pancreatic tumors, the mice are sacrificed, and the tumors are resected for FFPE. We employ FFPE processing to reduce the substantial water absorption loss associated with highly vascular fresh tissue specimens. For this study, we use 4 mm thick paraffin-embedded murine tissues to eliminate the possibility of drying bare tissue samples when exposed to ambient conditions, particularly the dry-nitrogen atmosphere during THz-TDS experiments. Two tumor tissue blocks, one with a healthy pancreas region at its boundary and one without any healthy pancreas region, are reported in this work. The samples are ∼400× thicker than the usual histopathology slides to avoid a Fabry–Perot etalon effect. Since the samples are thick enough to be free-standing, we can easily perform THz-TDS in a transmission geometry.^22,23^ In addition, we also section the tissues to 2 μm thick specimens for hematoxylin/eosin (H&E) staining. At the data analysis stage, THz images are aligned to sequential sections stained with H&E.

### 2.3 Frequency-Domain (FD) Parameter Estimation Technique

The most significant advantage of the THz-TDS method is that it can probe the sample’s contribution to both the amplitude and phase of the THz radiation, which makes it possible to evaluate the sample’s optical parameters without employing Kramers–Kronig relations. This is, however, a double-edged sword when it comes to extraction of the optical parameters. The standard FD approach is to take a Fourier transform of both the sample and reference pulses and use the sample spectrum normalized to the reference one to obtain the complex transmission or reflection coefficients based on the geometry of the experimental setup and, next, use it in the following equation:^24^
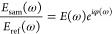
1where *E*_ref_ and *E*_sam_ are the Fourier transforms of the reference and the sample TD signals, respectively.

In our case of a thick sample the extinction coefficient ≪1, so ignoring the Febry–Perot terms, the complex transfer function can be written as the following approximated form using the Fresnel equation:^25^
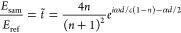
2where , , *d* stands for the sample thickness, *c* is the speed of light, and ω is the angular frequency. Hence, from eq 2, we can solve the inverse problem, where both *E*_sam_(ω) and *E*_ref_(ω) are known quantities from the experiment and we are looking for *n* and α. This problem is traditionally answered analytically, assuming optically thick samples (*nd* > 1.5 mm) and ignoring the phase term in the transmission coefficient and the losses during the pulse propagation.^21^ In this approach, one evaluates the real part of *n* by unwrapping the phase of *t̃*, which often introduces numerical instability.

Another approach taken in the literature is to solve this inverse problem iteratively by calculating *n* using the unwrapped phase and including an imaginary term to offset the loss difference.^26^ To do so, for each frequency, an error function with arbitrary weighting that contains both the modulus and phase error between the experimentally obtained and modeled transmission coefficients is minimized. Nonetheless, causality is not satisfied in this calculation, which ends up taking the shape of the Kramers–Kronig relation in the problem.^21^ This poses a substantial issue during the phase unwrapping because this is highly dependent on the dynamic range and, thus, is band limited. The latter is the principal drawback of the FD parameter extraction method, requiring further steps in phase unwrapping, since the phase gets lost beyond the dynamic range. The FD method is also limited by the arbitrary weighting of the phase and the amplitude in the error function. In literature it has been reported that the signals transmitted through the cancer tissue regions appear to be highly attenuated, posing a substantial challenge in phase unwrapping.^27^

### 2.4 Time-Domain (TD) Maximum *A Posteriori* Probability (MAP) Parameter Estimation Technique

To overcome the shortcomings of the FD parameter extraction approach, we propose to solve the inverse problem with full TD inversion. We consider the reference and the sample pulses, measured by THz-TDS, as a dynamical system. Hence, we adopt a transfer-function-based approach to extract the optical parameters.^24,25^ There are several advantages of solving the inverse problem in the TD with respect to FD. In TD, we can acquire high-SNR experimental signals for both the sample and the reference with femtosecond time resolution and very low noise, which helps us have more accurate estimates of the tissue *n* and α parameters. Therefore, we describe the estimation problem as the root-mean-square difference of the experimental sample signal and the modeled sample trace from the reference. Thus, we project this as a MAP estimation problem. Similar time-domain minimization strategies for inverse problems in medical imaging have been demonstrated in ultrasound elasticity imaging to provide reliable estimates in ultrasound-based rheological parameter inversion.^28,29^

In order to express the MAP estimator for the pulsed THz-TDS experiment, we traverse the reference pulse *E*_ref_ through a filter *K*_θ_, which parametrically simulates the effect of the THz propagation through a sample to generate a modeled sample pulse. Then, using the difference between the experimental sample pulse *E*_sam_ and the modeled sample pulse as an objective function, we optimize the filter parameters. We treat *K*_θ_ as a continuous function for the sake of convenience. In our proposed model, *K*_θ_ = Γ(n*(θ_*m*_), *E*_ref_, *E*_sam_), where n*(θ_*m*_) is the complex *n*, while θ_*m*_ denotes a subset of parameters that describe the system and is regulated by the type of the model applied. Thus, the exact expression of the filter function depends on the dielectric model under consideration. For simplicity, we chose a Fresnel equation for a single dielectric layer in transmission geometry, so for a thick sample, *K*_θ_ takes the following form:
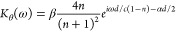
3where β is the amplitude scaling factor treated as Gaussian distribution of white noise, which is incorporated to compensate for variations in the reference pulse and the incident laser train amplitude fluctuations. We note that the pump beam power and low-frequency amplitude fluctuations are the major sources of noise in the THz-TDS system, and using the TD approach we can perform the noise modeling in a much simpler way, which is not possible in the FD analysis. The other benefit of the MAP method is that the filter function *K*_θ_ is modular in a sense that the system identification approach can be extended to include Fabry–Perot reflections and complex geometry conditions. For simplicity, in the present study, we use the Fresnel transfer function without the Fabry–Perot effect, but the same routine could be adapted for measurements in a reflection geometry, or studying multilayer samples, by simply changing the transfer function, or by adding the higher order Fabry–Perot terms.

The next step is the maximum likelihood estimation (MLE) process to best estimate the parameter values that transform *E*_ref_ to *E*_sam_, assuming uniform priors of *n* and α over the reconstructed parameters. We stress here that the amplitude scaling factor β is not an *a priori* known parameter but instead *a posteriori* derived. We then solve for the posterior probability of these parameters by using the time-domain minimization. For a particular point scan, knowing *E*_ref_ and *d*, we can construct the estimation problem in TD as the sum of mean-squared errors (MSEs) of the experimental trace and the modeled trace, shown as
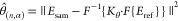
4

**Figure 4:**
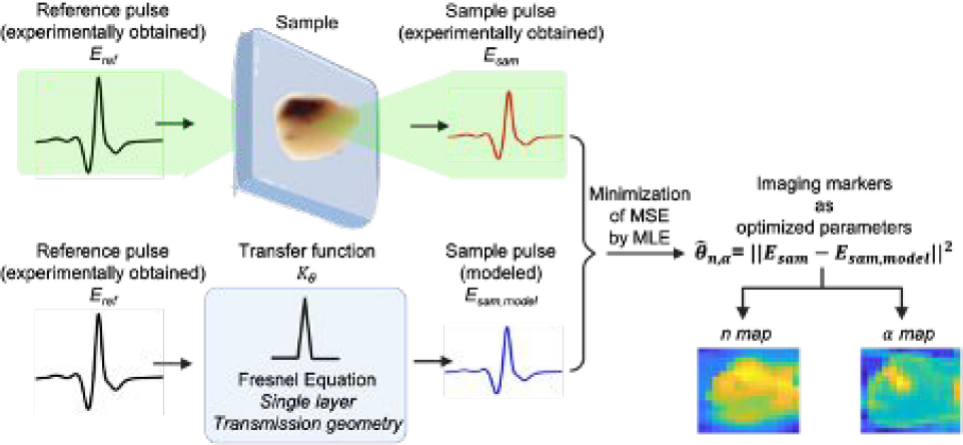
Block diagram of the MAP algorithm implemented in this study. A sample pulse *E*_sam_ is obtained from THz-TDS experiment for one scan point. A transfer function *K*_θ_ is designed based on the Fresnel transmission coefficient for a single-layer approximation to reconstruct a parametrically modeled pulse *E*_sam,model_. A minimization algorithm of the two pulses, i.e., *E*_sam_ and *E*_sam,model_, is performed using the MSE method in order to extract the optimized values of *n* and α. The process is repeated for all scan points to generate a THz map of the sample using the optimized *n* and α as imaging markers. Created with BioRender.com, agreement no. OV24VWZD5O.

The right-hand term of eq 4 is the difference between an experimental *E*_sam_(*t*) pulse and the *E*_sam,model_(*t*) one, reconstructed in TD from *E*_ref_. We first transform E_ref_(*t*) to its frequency-equivalent *^E*_*ref*_ (ω) using a temporal FFT operator *F* and multiply it by the impulse-function, which in our case is the Fresnel operator *K*_θ_(ω) defined in eq 3. Next, we transform the *K*_θ_(ω)*^E*_ref_ (ω) product back to TD using the inverse *F*^–1^ operator and, finally, compute the  norm of the residual. MLE yields estimations of the Fresnel parameters θ̂_*n*,α_, so the problem reduces to a two-parameter model and these parameters driving the impulse transform are the optical parameters of interest, namely, *n* and α [θ_*m*_ → θ_*m*_(*n*,α)]. A block diagram of the MAP algorithm is presented in Figure 4.

The MLE reconstruction of a pulse transmitted through our tissue sample is mathematically implemented using a nonlinear least-squares routine in MATLAB (R2021a). In this work, we select a simple gradient-free Nelder–Mead optimization algorithm because of its robustness and easy convergence.^30^ This method calculates a new error function (the  norm) at each iteration based on the current values of the parameters of interest and allows solving the problem for determining a parameter update by completing each iteration. This method gives L1-regularized estimates that can effectively deal with zeroes or large numbers in the solving equation. Since the most optimization routines are sensitive to the prior values in order to achieve the global minima, it is very important to select initial parameters that are close to the globallyoptimized parameters. For this, we use the prior values of *n* and α calculated from the regular FD analysis at their center frequency. After the optimization is completed, we register the optimized values of *n* and α, which are the average values within the usable frequency range, and use them as markers for differentiation of the studied sample characteristics to ensure reproducibility of findings and subsequent biological significance.

## 3 Results

**Figure 5:**
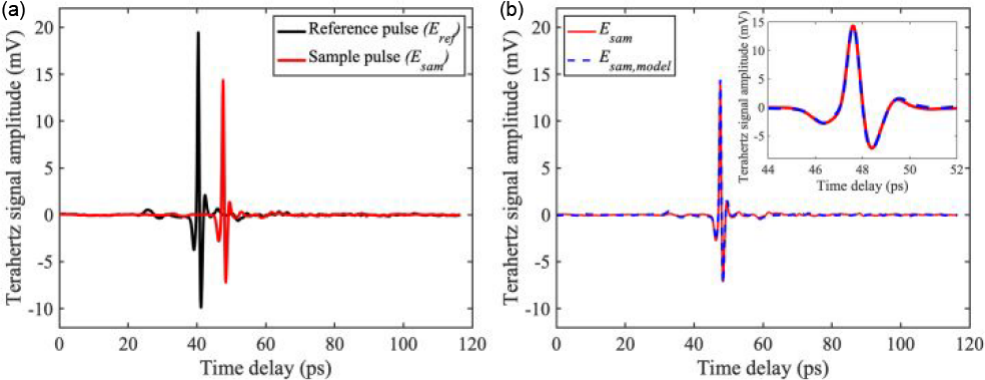
(a) THz time-domain traces of the reference *E*_ref_ (empty setup) and sample *E*_sam_ signals that were obtained experimentally. (b) The modeled time-domain signal constructed using MAP (*E*_sam,model_) overlaps with the experimentally obtained THz-TDS sample trace (*E*_sam_) (red line the same as that in Figure 5(a)) for the same sample. An overlap of the two signals is confirmed by the inset in (b), which shows a zoomed-in view of *E*_sam,model_ and *E*_sam_ close to the main peak position.

Figure 5(a) shows THz time-domain experimental transients measured in our THz-TDS system [Figure 2(a)]. The black trace corresponds to an empty setup and is denoted as *E*_ref_, while *E*_sam_ (red trace) is the signal transmitted through a sample. As expected, the signal transmitted through the test sample is attenuated and shifted in time, as compared to *E*_ref_. Figure 5(b) compares the experimental *E*_sam_ trace, the same as that in Figure 5(a), and the modeled sampled trace *E*_sam,model_ (dashed blue line) using MAP. We note that the sample pulse from the experiment and the modeled pulse overlaps, what is clearly visible in Figure 5(b) inset. Calculations of *E*_sam,model_ include the scaling factor β (see eq 3) that takes care of the noise effect and the algorithm described in section 2.4 converged at a numerical tolerance of 1e^–8^.^21^ The test result presented in Figure 5(b) demonstrates that using the MAP approach to solve the inverse problem in THz-TDS studies indeed provides an excellent convergence without any spurious artifacts.

### 3.1 MAP PDAC Imaging: Transverse and Longitudinal Scans

**Figure 6:**
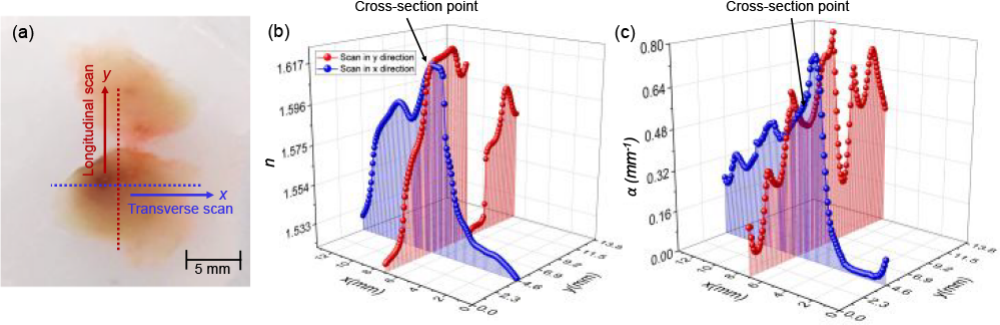
Transverse (*x*-direction) and longitudinal (*y*-direction) line scans of a paraffin-embedded pancreas tissue with PDAC. (a) Optical micrograph with scan lines indicated. (b) Refractive index values across the scan lines obtained using our MAP technique. (c) Absorption coefficient values across the scan lines obtained using our MAP technique. At the cross-section points (see arrows), the imaging markers’ values overlap, ensuring the repeatability of the measurement.

We made two cross-sectional scans on a 4 mm thick paraffin-embedded PDAC tissue with the 100 μm step size in both transverse (*x*) and longitudinal (*y*) directions [see also Figure 3(g)], shown in Figure 6(a) as blue and red dashed lines superimposed on an optical image of the studied tissue. The position and the length of each dashed line correspond to the position and length of the performed linear scans, respectively. Each scan point consists of a TD THz pulse with 1000 averages, and from each pulse we extracted the imaging markers, *i.e.*, *n* and α, using the MAP estimation technique. Next, we mapped out values of these parameters on two cross-sectional scans of the sample, presented as three-dimensional plots shown in Figure 6(b) and (c) for *n* and α, respectively. We notice that the tissue edges are well resolved, especially in the *y*-scan, when *n* drops sharply at a narrow paraffin region between two tissue nodules [see also Figure 6(a)]. The THz refractive index of the paraffin found by the MAP technique closely matches the literature value.^31^ The values of *n* and α changing within the tissue regions can help unveil changes in the tissue microenvironment, such as normal/tumor tissue boundaries, by tracing the corresponding values. Finally, at intersection points between the *x*- and *y*-scans, indicated by arrows in both Figure 6(b) and (c), the values of *n* and α, respectively, match precisely with each other. We have an additional confirmation that the proposed MAP parameter extraction method gives unbiased and reproducible results, since the *y*-scan is taken around 5 h after the *x*-scan. Hence the exact same values of the two imaging markers at the intersection points indicate the long-term stability and unbiases of the method, as *n* and α are both physical parameters unlike peak amplitude and spectral peak, which are heavily dependent on the stability of the THz-TDS system, especially the stability of the laser power.

### 3.2 THz-TDS MAP PDAC Imaging: Two-Dimensional Raster Scans

**Figure 7:**
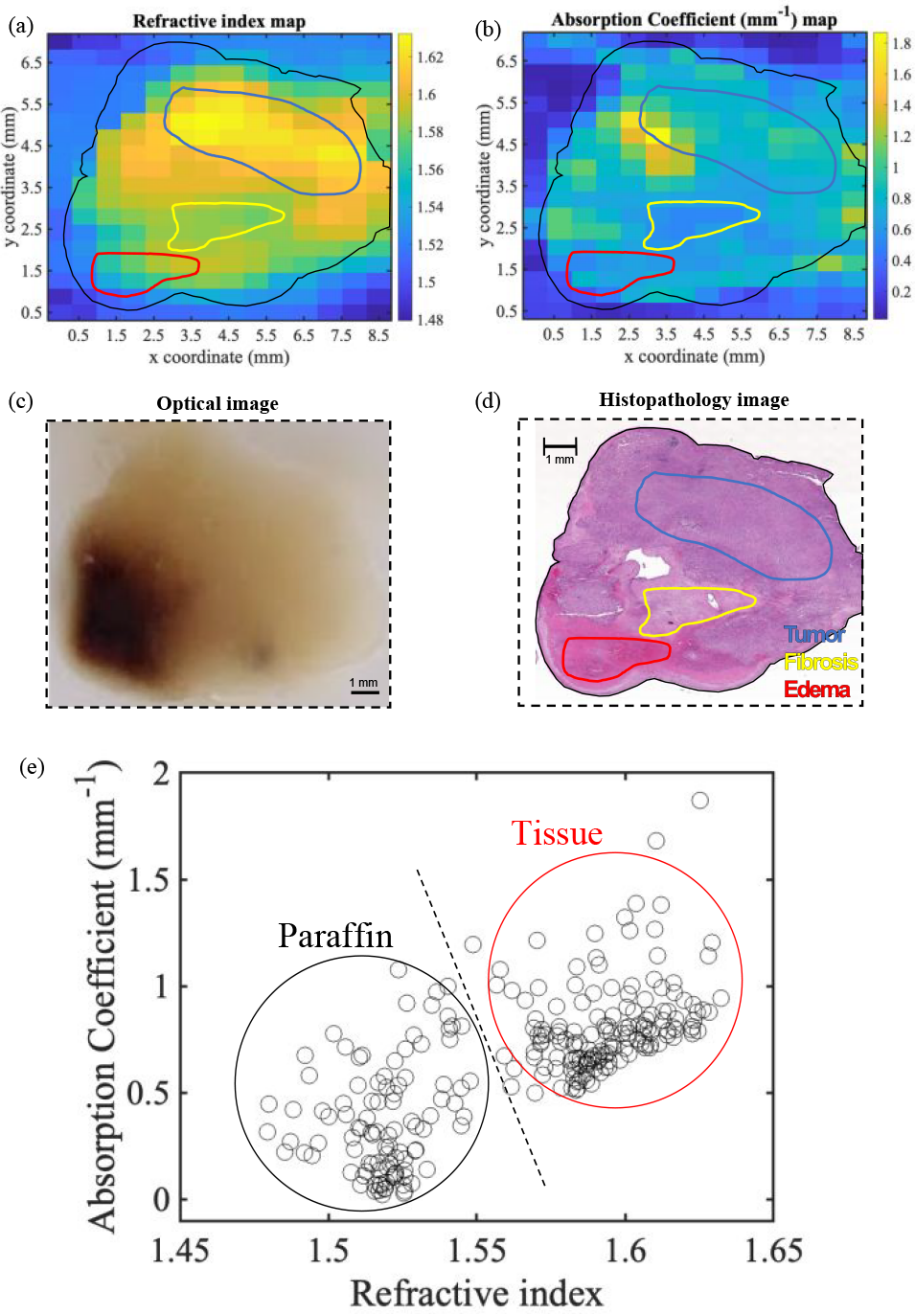
Two-dimensional images of (a) the refractive index and (b) the absorption coefficient, the THz imaging markers extracted using MAP for a paraffin-embedded PDAC tissue. (c) Optical image of the tested sample with the area corresponding directly to the size of the scans. (d) H&E-stained histopathology image of the sample with different regions indicated. (e) Plot of absorption coefficients of two imaging markers vs refractive index, showing two different clusters for the paraffin and the tissue regions.

Panels a and b in Figure 7 present two-dimensional (2D) images of a paraffin-embedded PDAC-only tissue using our optimized imaging markers, namely, *n* and α, respectively. For completeness, we also added in Figure 7 an optical image of the studied PDAC tissue [Figure 7(c)] and the corresponding histopathological image [Figure 7(d)]. Because of the manual *x*–*y* stage movement used in the experiment, the tissue was scanned with a 500 μm long step size; hence, the maps are highly pixelated. However, the tissue edges are clearly resolved. To assess the efficacy of THz imaging in mapping different features, bulk regions of tumor, fibrosis, and edema were defined using H&E-stained tissue sections [Figure 7(d)], and annotations were translated to refractive index [Figure 7(a)] and absorption coefficient [Figure 7(b)] maps generated from THz imaging on a neighboring section. It is well established that the composition of the PDAC tumor microenvironment is highly heterogeneous. The density and spatial distribution of several features can vary greatly between tumors, including regions of tumor, necrosis, fibrosis, immune infiltrate, and edema.^32^ It is worth mentioning that histopathology employed a much greater resolution to study the tissue with better precision, allowing for a closer examination at the cellular level to determine the distinct subregions within the tissue.^13^ The THz maps show that the dark blue areas in Figure 7(a and b) represent *n* and α of pure paraffin, respectively, whereas the “warmer” colors indicate the denser (higher *n*) and more absorbent (higher α) regions. It can be noted that within the tissue region a highly heterogeneous structure can be identified in both *n* and α maps. Comparing the H&E-stained histopathology image [Figure 7(d)] with the refractive index map [Figure 7(a)], we note that the tumor region exhibits an average *n* of 1.617 (±0.004), which is higher than those in the other regions identified as fibrosis and edema with average *n* values of 1.584 (±0.002) and 1.571 (±0.002), respectively. Upon comparing the histopathology map [Figure 7(d)] with the absorption coefficient map [Figure 7(b)], we note that the tumor region has a higher α value [1.011 mm^–1^ (±0.004)] compared to those of fibrosis, [0.667 mm^–1^ (±0.002)] and edema [0.664 mm^–1^ (±0.005)]. Overall, the tumor regions appear to be “warmer” because of the higher refractive index and absorption coefficient. The high *n* and α values of the tumor region compared to the other subregions is consistent with the trend reported in the literature for other types of malignancies such as breast tumor models.^27^ An optical tissue image [Figure 7(c)] has a dark spot in the bottom left side, which is due to tissue hemorrhage. This spot has no correlation to either the H&E-stained image or the THz maps. Finally, Figure 7(e) shows the *n* vs α plot, and we note that the imaging markers form two distinct clusters, namely, the paraffin and the tissue ones.

## 4 Discussion

**Figure 8:**
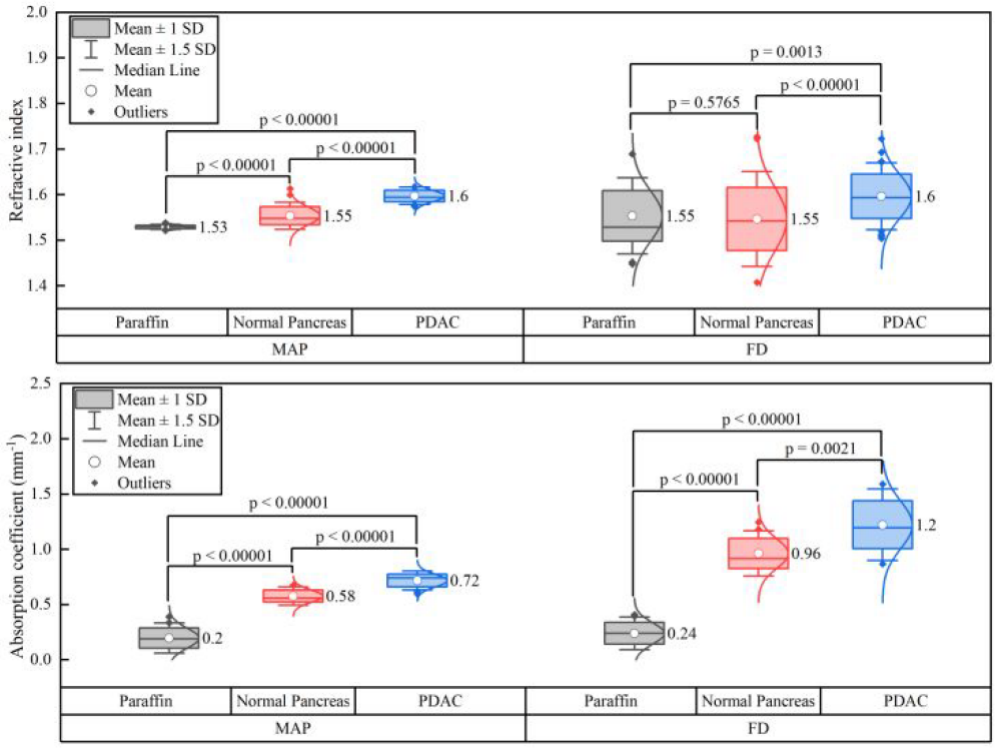
Boxplots showing the comparison of optimized THz imaging markers extracted from MAP (left side of the plot) and FD (right side of the plot) extraction methods, averaged over 50 scan points per trace for pure paraffin, normal pancreas, and PDAC within the same tissue sample under examination, with the *p*-values indicated for the 1000 measurement average for the refractive index *n* (upper panel) and the absorption coefficient α (lower panel). The 25th and 75th percentiles are represented by the lower and upper box boundaries, respectively, and the median value is indicated by the straight line inside each box. Next to each box is the respective mean values. Each box also has the data’s Gaussian distribution added next to it.

For completeness of our work, we calculated and present here a comparison between the imaging markers obtained using MAP and FD techniques for paraffin-embedded normal and PDAC regions in a form of boxplot representation, as shown in Figure 8. It is clearly visible that both imaging markers obtained from MAP exhibit a narrower standard deviation as compared to those obtained from FD analysis, implying that MAP gives a significantly more robust estimation of the imaging markers. In case of MAP, the three material groups, *i.e.*, pure paraffin, normal tissue, and PDAC, exhibit statistically significant values with *p* < 0.00001 in both the cases of *n* and α. As a result, as these two parameters *n* and α obtained using MAP can offer statistically significant estimates, we suggest using them as imaging markers for identifying tissue regions in extremely complex and heterogeneous biological microenvironments.

Table 1, in turn, compares our MAP-based *n* and α parameters to other imaging markers reported in the literature to present the tissue characteristics for tumor imaging with THz-TDS. Again, we present the results based on the characterization of our paraffin-embedded pancreas tissues. Each row corresponds to a particular sample group (paraffin, normal pancreas, and PDAC), while each column corresponds to a particular feature or an imaging marker. Since different imaging markers have different units, we chose to look at the coefficient of variation (CV) [Table 1(a)] defined as the ratio between standard deviation and mean value. The CV does not depend on the dimension or unit of the imaging markers. In addition, in Table 1(b), we present the interquartile range (IQR) defined as the difference in the 25th and 75th percentiles of a given data set.

**Table 1:**
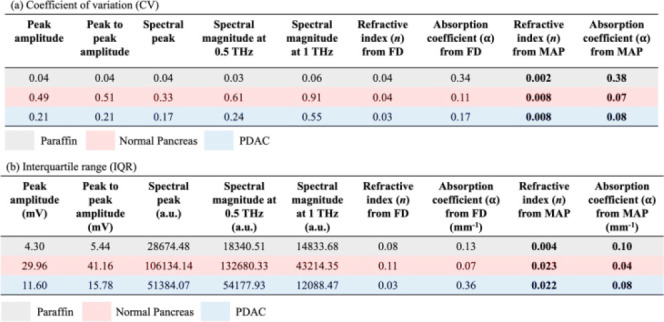
(a) Coefficient of Variation (CV) and (b) Interquartile Range (IQR) for the Common Features Used over the Literature along with the MAP-Extracted Parameters for Paraffin, Normal Tissue, and PDAC Regions

Table 1(a) shows that *n* obtained from MAP has the lowest CV for a particular sample group. The value is approximately two orders of magnitude smaller than the rest of the features, ensuring that the spread of data points for *n* from the MAP is low compared to its mean value. The CV for the *n* from FD has a larger value due to the inconsistent values extracted from FD due to the phase unwrapping issue. The second-lowest value of CV is obtained for α values extracted for MAP for the normal pancreas and PDAC, which means that the standard deviation is high compared to its mean value. CV for α has a higher value than the other markers, which might seem counterintuitive. However, the reason is that the standard deviation of α for paraffin is very low compared to its mean because paraffin is an inorganic sample, which is also evident from the boxplot presented in Figure 8. Therefore, we conclude that our proposed imaging markers *n* and α derived using the MAP approach are the best choice when compared to traditional imaging markers, since the lower the CV value means the more precise estimate of the marker.

Table 1(b) demonstrates that the IQR follows a similar trend to CV, *i.e.*, the IQR has its lowest value for *n* and α extracted from MAP, when compared to the other markers, ensuring less dispersion in the data points while classifying normal and tumor regions within the pancreas. As a result, the THz properties *n* and α of materials obtained through MAP provide the most conservative estimates. Hence, they are the excellent classifiers to differentiate the tumor and healthy regions in the pancreatic tissue.

## 5 Conclusion

In conclusion, we established a set of imaging markers, *n* and α, by performing the MAP estimation process on experimental THz transients collected using the THz-TDS technique. We have demonstrated that this MAP-based THz parameter extraction pipeline can effectively return THz-regime parameters of the tissue by only knowing TD THz traces that uniquely map characteristics of the sample-under-investigation. We validated the effectiveness of our algorithm by performing cross-sectional line scans of PDAC as well as normal tissue samples encapsulated in paraffin. We extrapolated our method to achieve 2D raster scans of a pancreatic tissue sample with different anatomical regions to show that even subtle changes in the tissue microenvironment markedly impacted the tissue optical properties in the THz range. We can map those changes using the markers extracted from the MAP. Thus, our work intends to establish standardized imaging markers for THz imaging of PDAC tissue to enable a reproducible and unbiased analysis of THz-TDS measurements. Our mathematical approach should be valid for any tissue samples studied using the transient THz spectroscopy method. This work demonstrates applicability of the THz-TDS imaging method for examination of subregions of a complex tumor case such as pancreatic ductal of adenocarcinoma and shows the potential of THz imaging as an *ex vivo* imaging platform for objectively mapping tumor responses to immunotherapy treatments. One potential limitation of this work is that we need to select initial parameters close to the global optimized parameters to achieve global minima. Another limitation is that we have to evaluate the parameters for each pixel from a TD trace taken for that pixel, making the entire evaluation and image mapping computationally heavy and time consuming. We intend to address these issues in our future work by employing more cost- and time-efficient methods, by, e.g.,parallelizing our code.

In the presented work, the THz line-scans and THz maps were generated by manually moving the *x*–*y* stage and, subsequently, saving the signal information at each pixel location. Our future work is directed toward building an automated translational stage system with reduced scan step size to improve the spatial resolution as well as increase the data acquisition speed. This will enable us to produce high-resolution, large-area THz images that can be used to identify tissue subregions with clearly defined borders between them. Our future work will also include THz path modeling through pancreatic tissue with complex heterogeneity to probe the tumor microenvironment’s subtleties by understanding the nature of THz interaction with the tissue. Additionally, we will work on substrate tissue matching, as the substrate plays the crucial role in the reconstruction of the THz imaging markers of the tissues. In conclusion, our experiments were conducted utilizing a commercial large-footprint Ti:sapphire laser on an optical table. It is worth noting that alternative compact femtosecond fiber lasers are commercially available. Utilizing these lasers, the entire THz-TDS system has the potential to be designed as a compact, portable unit.
